# Supplementary material for: A Critical View of the Application of the APEX Software (Aqueous Photochemistry of Environmentally-Occurring Xenobiotics) to Predict Photoreaction Kinetics in Surface Freshwaters
Source: Molecules. 2019 Dec 18;25(1):9. doi: 10.3390/molecules25010009 (PMC7017383; doi:10.3390/molecules25010009)
Supplement: Supplementary file 1 [file molecules-25-00009-s001.zip › 3.2.4_gcc-4.4.0.zip!/3.2.4_gcc-4.4.0/3.2.4_gcc-4.4.0/doc/octave/html/interpreter/Documentation-Tips.html]

Documentation Tips - Untitled


Previous: Function Headers,
Up: Tips and Standards


---

### C.5 Tips for Documentation Strings

As noted above, documentation is typically in a commented header block
on an Octave function following the copyright statement. The help string
shown above is an unformatted string and will be displayed as is by
Octave. Here are some tips for the writing of documentation strings.

- Every command, function, or variable intended for users to know about
  should have a documentation string.- An internal variable or subroutine of an Octave program might as well have
    a documentation string.- The first line of the documentation string should consist of one or two
      complete sentences that stand on their own as a summary.

      The documentation string can have additional lines that expand on the
      details of how to use the function or variable. The additional lines
      should also be made up of complete sentences.- For consistency, phrase the verb in the first sentence of a
        documentation string as an infinitive with “to” omitted. For
        instance, use “Return the frob of A and B.” in preference to “Returns
        the frob of A and B.” Usually it looks good to do likewise for the
        rest of the first paragraph. Subsequent paragraphs usually look better
        if they have proper subjects.- Write documentation strings in the active voice, not the passive, and in
          the present tense, not the future. For instance, use “Return a list
          containing A and B.” instead of “A list containing A and B will be
          returned.”- Avoid using the word “cause” (or its equivalents) unnecessarily.
            Instead of, “Cause Octave to display text in boldface,” write just
            “Display text in boldface.”- Do not start or end a documentation string with whitespace.- Format the documentation string so that it fits in an Emacs window on an
                80-column screen. It is a good idea for most lines to be no wider than
                60 characters.

                However, rather than simply filling the entire documentation string, you
                can make it much more readable by choosing line breaks with care.
                Use blank lines between topics if the documentation string is long.- **Do not** indent subsequent lines of a documentation string so
                  that the text is lined up in the source code with the text of the first
                  line. This looks nice in the source code, but looks bizarre when users
                  view the documentation. Remember that the indentation before the
                  starting double-quote is not part of the string!- The documentation string for a variable that is a yes-or-no flag should
                    start with words such as “Nonzero means...”, to make it clear that
                    all nonzero values are equivalent and indicate explicitly what zero and
                    nonzero mean.- When a function's documentation string mentions the value of an argument
                      of the function, use the argument name in capital letters as if it were
                      a name for that value. Thus, the documentation string of the operator
                      `/` refers to its second argument as DIVISOR, because the
                      actual argument name is `divisor`.

                      Also use all caps for meta-syntactic variables, such as when you show
                      the decomposition of a list or vector into subunits, some of which may
                      vary.

Octave also allows extensive formatting of the help string of functions
using Texinfo. The effect on the online documentation is relatively
small, but makes the help string of functions conform to the help of
Octave's own functions. However, the effect on the appearance of printed
or online documentation will be greatly improved.

The fundamental building block of Texinfo documentation strings is the
Texinfo-macro `@deftypefn`, which takes three arguments: The class
the function is in, its output arguments, and the function's
signature. Typical classes for functions include `Function File`
for standard Octave functions, and `Loadable Function` for
dynamically linked functions. A skeletal Texinfo documentation string
therefore looks like this

```
     -*- texinfo -*-
     @deftypefn{Function File} {@var{ret} =} fn (...)
     @cindex index term
     Help text in Texinfo format.  Code samples should be marked
     like @code{sample of code} and variables should be marked
     as @var{variable}.
     @seealso{fn2}
     @end deftypefn
```

This help string must be commented in user functions, or in the help
string of the `DEFUN_DLD` macro for dynamically loadable
functions. The important aspects of the documentation string are

-\*- texinfo -\*-: This string signals Octave that the following text is in Texinfo format, and should be the first part of any help string in Texinfo format. @deftypefn{class} ... @end deftypefn: The entire help string should be enclosed within the block defined by deftypefn. @cindex index term: This generates an index entry, and can be useful when the function is included as part of a larger piece of documentation. It is ignored within Octave's help viewer. Only one index term may appear per line but multiple @cindex lines are valid if the function should be filed under different terms. @var{variable}: All variables should be marked with this macro. The markup of variables is then changed appropriately for display. @code{sample of code}: All samples of code should be marked with this macro for the same reasons as the @var macro. @seealso{function2}: This is a comma separated list of function names that allows cross referencing from one function documentation string to another.

Texinfo format has been designed to generate output for online viewing
with text terminals as well as generating high-quality printed output.
To these ends, Texinfo has commands which control the diversion of parts
of the document into a particular output processor. Three formats are
of importance: info, html and TeX. These are selected with

```
     @ifinfo
     Text area for info only
     @end ifinfo
```

```
     @ifhtml
     Text area for HTML only
     @end ifhtml
```

```
     @tex
     Text area for TeX only
     @end tex
```

Note that often TeX output can be used in html documents and so often
the `@ifhtml` blocks are unnecessary. If no specific output
processor is chosen, by default, the text goes into all output
processors. It is usual to have the above blocks in pairs to allow the
same information to be conveyed in all output formats, but with a
different markup. Currently, most Octave documentation only makes a
distinction between TeX and all other formats. Therefore, the
following construct is seen repeatedly.

```
     @tex
     text for TeX only
     @end tex
     @ifnottex
     text for info, HTML, plaintext
     @end ifnottex
```

Another important feature of Texinfo that is often used in Octave help
strings is the `@example` environment. An example of its use is

```
     @example
     @group
     @code{2 * 2}
     @result{} 4
     @end group
     @end example
```

which produces

```
     2 * 2
     => 4
```

The `@group` block prevents the example from being split across a
page boundary, while the `@result{}` macro produces a right
arrow signifying the result of a command. If your example is larger than
20 lines it is better NOT to use grouping so that a reasonable page boundary
can be calculated.

In many cases a function has multiple ways in which it can be called,
and the `@deftypefnx` macro can be used to give alternatives. For
example

```
     -*- texinfo -*-
     @deftypefn{Function File} {@var{a} =} fn (@var{x}, ...)
     @deftypefnx{Function File} {@var{a} =} fn (@var{y}, ...)
     Help text in Texinfo format.
     @end deftypefn
```

Many complete examples of Texinfo documentation can be taken from the
help strings for the Octave functions themselves. A relatively complete
example of which is the `nchoosek` function. The Texinfo
documentation string for `nchoosek` is

```
     -*- texinfo -*-
     @deftypefn {Function File} {} nchoosek (@var{n}, @var{k})
     
     Compute the binomial coefficient or all combinations of
     @var{n}.  If @var{n} is a scalar then, calculate the
     binomial coefficient of @var{n} and @var{k}, defined as
     
     @tex
     $$
      {n \choose k} = {n (n-1) (n-2) \cdots (n-k+1) \over k!}
     $$
     @end tex
     @ifnottex
     
     @example
     @group
      /   \
      | n |    n (n-1) (n-2) ... (n-k+1)
      |   |  = -------------------------
      | k |               k!
      \   /
     @end group
     @end example
     @end ifnottex
     
     If @var{n} is a vector, this generates all combinations
     of the elements of @var{n}, taken @var{k} at a time,
     one row per combination.  The resulting @var{c} has size
     @code{[nchoosek (length (@var{n}),@var{k}), @var{k}]}.
     
     @code{nchoosek} works only for non-negative integer arguments; use
     @code{bincoeff} for non-integer scalar arguments and for using vector
     arguments to compute many coefficients at once.
     
     @seealso{bincoeff}
     @end deftypefn
```

which demonstrates most of the concepts discussed above.
